# *IUCrData* launches Raw Data Letters

**DOI:** 10.1107/S2414314622008215

**Published:** 2022-08-23

**Authors:** L. M. J. Kroon-Batenburg, J. R. Helliwell, J. R. Hester

**Affiliations:** a Department of Chemistry, Structural Biochemistry, Bijvoet Centre for Biomolecular Research, Faculty of Science, Utrecht University, Utrecht, The Netherlands; b Department of Chemistry, The University of Manchester, Manchester M13 9PL, United Kingdom; c Australian Nuclear Science and Technology Organisation, Locked Bag 2001, Kirrawee DC, NSW 2232, Australia

**Keywords:** Raw Data Letters, imgCIF

## Abstract

A new category of articles – Raw Data Letters – is introduced to *IUCrData*.

*IUCrData*, the peer-reviewed open-access data publication from the International Union of Crystallography (IUCr), is launching a new section – Raw Data Letters. This is a collaborative innovation of IUCr Journals with the IUCr Committee on Data. Future raw data sets will become increasingly large and no one group will be able to analyze all of the scientific content in a timely manner. As already occurs in other scientific disciplines (*e.g.* astronomy, particle physics) others will need to have access to raw crystallographic data sets so that open-science-based research can proceed at a rapid pace. However, proper credit needs to be attributed fairly among those who design experiments and collect data, and those who subsequently use the data to establish new results.

With these points in mind, the new section will publish short descriptions of crystallographic raw data sets from X-ray, neutron or electron diffraction experiments, in the biological, chemical, materials science or physics fields, and provide a persistent link to the location of the raw data. The letters in this section will describe interesting features in raw data sets, allowing researchers to attract attention to particular aspects of the data that could be of interest to methods and software developers for purposes such as reanalysis by newer methods or may be relevant to the structural interpretation. We envisage different types of Raw Data Letters. The structure could have been solved and published elsewhere, but the letter describes interesting features that were observed but ignored in the original structure determination. The letter could describe remarkable features but no attempt is made as to their interpretation; in this way the data attract attention and the original authors get credit for their work. The raw data described in a letter show Bragg reflections to a reasonable resolution but the structure could not be solved. Again the authors would get credit for their work. Also letters describing the reuse of publicly available data by improved methods are welcome. In general, publication in Raw Data Letters promotes data retrieval by other scientists and will enhance visibility of the data. Raw Data Letters support Open Science policies: no research data should be lost but should be made available to the research community according to the FAIR principles, for which the correctness and completeness of the metadata are crucial, and these will be central to the reviewing process.

Science funders and policy makers are working increasingly towards the Open Science model to make science useful for society. Good scholarship demands openness and transparency of protocols and scientific results as well as proper data management and validation of scientific knowledge. Many open science platforms have seen the light, *e.g*. the OpenAIRE project (https://www.openaire.eu) and the European Open Science Cloud (EOSC, Jones, 2015 ▸) promoting the sharing of data. Guidelines for proper data management are described in *The FAIR principles for scientific data management and stewardship* by Wilkinson *et al.* (2016 ▸), which requires research data to be Findable, Accessible, Interoperable and Reusable.

What does this mean for crystallographic data?

*Findable*: the data are assigned a persistent identifier, they come with metadata by which they are indexed in a searchable resource.

*Accessible*: the data should be retrievable through well established communication protocols.

*Interoperable*: the data use a shared (documented) broadly applicable language for knowledge representation.

*Reusable*: the metadata should accurately describe experimental attributes of the data, and are released with a clear and accessible data usage license. Data formats should be described.

The IUCr has always taken a leading position in data sharing by linking publications to coordinates and structure factors as well as validation reports. In chemical crystallography the checkCIF tool, as part of the submission system, ensures consistency and integrity of the data. Likewise, in macromolecular crystallography a paper describing a crystal structure has to link to the Protein Data Bank (PDB) entry, while the wwPDB deposition system generates a validation report. The IUCr established a working group in 2011 (DDDWG) to address ‘the growing calls within the crystallographic community for the deposition of primary diffraction images, with some mechanism that allows their retrieval by other scientists for such purposes as reanalysis, software and methods development, validation and review’ (see https://forums.iucr.org/). The final report of the DDDWG made a series of recommendations, the first two of which are as follows.

Authors should provide a permanent and prominent link from their article to the raw data sets which underpin their journal publication and associated database deposition of processed diffraction data (*e.g.* structure factor amplitudes and intensities) and coordinates, and which should obey the ‘FAIR’ principles, that their raw diffraction data sets should be Findable, Accessible, Interoperable and Re-usable (https://www.force11.org/group/fairgroup/fairprinciples).

A registered Digital Object Identifier (DOI) should be the persistent identifier of choice (rather than a Uniform Resource Locator, URL) as the most sustainable way to identify and locate a raw diffraction data set.

The coordinating and advisory role of the DDDWG has been continued by the Committee on Data (CommDat), which was established by the IUCr in 2016. This committee plays a significant role in the current initiative.

A series of papers appeared in *Acta Cryst. D* in 2014 (Guss & McMahon, 2014 ▸; Kroon-Batenburg & Helliwell, 2014 ▸; Terwilliger & Bricogne, 2014 ▸; Meyer *et al.*, 2014 ▸) discussing the possibilities of raw-data depositions either in centralized facilities or distributed repositories. Whereas at that time the possibilities of data transfer and estimated costs of storage and curation were seen as a barrier, in recent years several freely accessible repositories have become available that make routine raw-data deposition feasible, and the bandwidth of internet connections has also increased substantially. A recent editorial from IUCr journals (*FAIR diffraction data are coming to protein crystallography*, Helliwell *et al.*, 2019 ▸) encourages authors to provide a DOI for their original raw data when submitting their article.

In a topical review in *IUCrJ* (Kroon-Batenburg & Helliwell, 2014 ▸), the requirements for metadata are discussed. Without correct and complete metadata we would certainly not adhere to the FAIR principles as the reusability would be compromised.

We also note and have warmly welcomed the PDBj initiative in 2021 to launch its own raw diffraction data archive (https://xrda.pdbj.org/), which is integrated with PDBj, and which opens up the FAIR principles and data record right back to the time of measurement in a depositor’s research studies.

With Raw Data Letters we want to elicit re-use of the original data. Diffraction data can be from various disciplines: macromolecular crystallography, chemical crystallography, XFELs, synchrotron serial crystallography, materials science powder diffraction *etc*. These come with many different data formats and varying metadata quality. To ensure reusability, metadata should be accurate and complete and at least sufficient. For single-crystal data we have made a list of core metadata; their presence is a key requirement for correct (automated) reprocessing of the data. Our list is a superset of the NeXus/HDF5 NxMx Gold Standard that was developed by Bernstein *et al.* (2020 ▸). We decided to capture metadata in imgCIF (Bernstein & Hammersley, 2005 ▸; Hammersley *et al.*, 2005 ▸), which is well known to the community via its CBF variant, and already includes the appropriate data names for our core metadata list (see Table 1 ▸). imgCIF also has the advantage of being a plain-text format, which allows editing with familiar tools and provides excellent guarantees of readability over the long term.

The current core metadata list has been specifically developed for single-crystal data; it will have to be extended for powder diffraction and high-pressure data; XFEL data will also need additional information.

There are two aspects to metadata that need attention after having established the core metadata list: (1) we need to generate the metadata from the original raw data format, possibly completed by user-supplied metadata, and (2) we need a checking procedure for checking consistency and correctness that can be used on the IUCr webserver.

A separate working group has been developing tools for extracting metadata information from raw images. A key problem is that raw data, unlike structural data, may be deposited in one of a multitude of formats currently in use. The situation for large-scale facilities is somewhat different to that for home diffractometers. Large-scale facilities often have PILATUS detectors that use CBF binary data format and miniCBF (ASCII) headers or EIGER detectors that use the Nexus/HDF5 data structure; at the same time CCD detectors may still be in use. Home diffractometers often have manufacturer-developed detectors and binary image formats. Most home diffractometers also have multi-circle diffractometers that make the description of metadata more complicated.

Practical issues have forced us to compromise on the DDDWG recommendations above. We have adopted an approach where the metadata, in the form of an imgCIF file, is separated from the raw data frames, to avoid the need for unfeasibly large data downloads during the validation process. The imgCIF file required during submission instead contains internal pointers to the raw data as URLs. The use of URLs, rather than more robust DOIs, is an additional compromise resulting from the requirement that checks be able to access the actual data files containing the data frames; there is no standardized way of finding the data files from data DOIs as the DOI usually resolves to an informational ‘landing page’. To mitigate against the URL fragility that DOIs were designed to avoid, the accessibility of all repositories referenced by archived imgCIF files held by the journal will be regularly checked, and in the rare case that URLs become inaccessible they will be manually updated using the data DOI provided by the authors during submission. Once the data DOI specifications mature to the point that data files can be directly referenced, our approach envisages that the URL form of a data DOI would be used in the submitted imgCIF file. We understand that our solution is necessarily a compromise, and will continue to refine the way in which raw data are handled as we gain experience and receive feedback from the community.

For the first letters published, we generated complete and consistent imgCIF files using the CBF laboratory axis system. For the macromolecular letter (Neviani *et al.*, 2022 ▸), we used a Python script written by Fabio Dall’Antonia and Julian Hörsch (European XFEL) to convert the metadata information from a Nexus/HDF5 master file into CBF/imgCIF. This particular Diamond Light Source Nexus/HDF5 (meta)data follows the ‘Gold Standard’ defined by the HDRMX working group (Bernstein *et al.*, 2020 ▸). The CBF laboratory axis system is different from that of Nexus McStas. Fig. 1 ▸ explains the details.

Most diffractometer manufacturers provide software for conversion to CBF, mostly miniCBF headers and CBF binary data. Bruker AXS was very helpful in providing a tool for image conversion to full CBF files. We used these files as the basis for our imgCIF file in the letter describing the twinned form of *o*-nitro­aniline (Lutz & Kroon-Batenburg, 2022 ▸).

Procedures and tools for the generation of imgCIF files from the available image data files are being incorporated into the IUCr Journals submission system. This is ongoing work, as we have to deal with many different formats, and contributions from the community are welcome. Further information on tools for authors will available shortly.

Submissions are now being welcomed – updated Notes for authors and submission instructions are available from the *IUCrData* website. This is early days for the Raw Data Letters section. Our Co-editors (see https://iucrdata.iucr.org/x/services/editors.html) are very keen to work with authors to facilitate publication of their data. We look forward to receiving your Raw Data Letters.

## Figures and Tables

**Figure 1:**
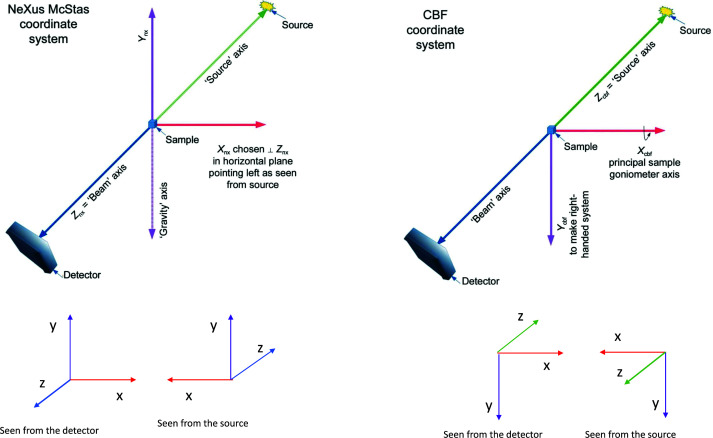
The Nexus McStas axis system is different from the CBF laboratory system. Seen from the source in McStas, *Z* points away from the source and the *X* axis points to the left, while in CBF *Z* points back into the source and *X* is along the principal goniometer axis, *i.e.* points into the goniometer block. A vector defined in McStas is brought by a 180° rotation around Nexus_X to the CBF system. Note that if the goniometer is at the opposite side, the transformation is around Nexus_Y (image courtesy of the Gold Standard paper, Bernstein *et al.*, 2020 ▸).

**Table 1:** Core metadata list and their data names in imgCIF

Minimal metadata	imgCIF data name
Data binary format	_array_structure.byte_order, _array_structure_compression_type, _array_structure.encoding_type
	
Number of pixels, pixel size (binning mode)	_array_structure_list.index _array_structure_list.dimension _array_structure_list_axis.displacement_increment
	
Beam center (mm)	_axis.offset[1..3] (needs _axis category, see below) _diffrn_scan_frame_axis.displacement
	
Origin of data frame	_array_structure_list.precedence (1 or 2) _array_structure_list.direction (increasing or decreasing)
	
Wavelength	_diffraction_radiation_wavelength.value(/wavelength) or _diffraction_radiation.type
	
Rotation axis	_diffrn_scan_axis.axis_id _diffrn_scan_axis.angle_start _diffrn_scan_axis.angle_range
	
Rotation range per frame/number of frames	_diffrn_scan_axis.angle_increment,_diffrn_scan.frames _diffrn_scan_frame.frame_number
	
Axes and offsets	_axis.id, _axis.type, _axis.depends_on, _axis.vector[1] _axis.vector[2] _axis.vector[3] _axis.offset[1] _axis.offset[2] _axis.offset[3]
	
Detector-to-sample distance	_diffrn_scan_axis.displacement_start _diffrn_scan_axis.displacement_range
	
Conditions	_diffrn.ambient_temperature
